# Iranian neurological events: The 20^th^ Iranian Congress of Neurology and Electrophysiology

**Published:** 2013

**Authors:** Mansoureh Togha, Abdorreza Naser Moghadasi

**Affiliations:** 1 Professor, Department of Neurology, Sina Hospital, Tehran University of Medical Sciences, Tehran, Iran; 2 Neurologist, Sina MS Research Center, Sina Hospital, Tehran University of Medical Sciences, Tehran, Iran

The 20^th^ Congress of Neurology and Electrophysiology of Iran was held in Tehran from April 30 to May 03, 2013. The congress was supported by Iranian Neurological Association and about 600 physicians and researchers participated in the congress. Prof. Atri, Prof. Federico, Prof. Rostami, Prof. Lotfi, and Dr. Ansarinia, Dr. Salajegheh, Dr. Banikazemi, Dr. Sybilleh, and Dr. Nagga were the international guests of the congress.

The congress was managed by Prof. Togha and nicely supported by the president of the Neurological Association, Prof. Pakdaman. The main aim of the congress was to familiarize neurologists and other specialists with the latest scientific achievements in the field of neurology.

The interesting characteristics of this congress was that for the first time abstracts of lectures were available online beforehand and were published as a supplement of Iranian journal of neurology (2013; 12(supp 1)). This was admirably managed by Dr. Fatehi who is the deputy editor of Iranian Journal of Neurology.

Accordingly, several scientific programs were held during four days. The programs were divided into the following 6 categories:

1. ***Teaching courses***
  Neurologists who were considered expert in a specific field offered the latest instructions and information on prevalent and important diseases. The courses were mostly educational and discussed about the major areas of neurology.
2. ***Different clinical and research aspects of neurology***
  This section comprised 61 lectures about studies conducted by the lecturers. For the first time, one of the most important parts of this program was allocated to neurometabolic diseases. It was systematically designed by Dr. Shalbafan. Our special guest in this field was Prof. Federico from Italy who is the chief editor of Neurological Science Journal (the official journal of the Italian Neurological Society).
3. ***Workshops***
  In this section, scientific aspects of neurology were considered and younger neurologists were trained on techniques like electroencephalography (EEG), electromyography (EMG), transcranial color coded Doppler (TCCD), and botulinum injection protocol for chronic migraine. Furthermore, for the first time in an Iranian neurology congress, two special workshops were held about Medical Digital Library and how to study and review articles.
4. ***Panels***
  Two panels were held during the congress. The first panel discussed different aspects of multiple sclerosis and the other panel reviewed the aspects of stem cell therapy in neurological diseases.
5. ***Interesting and challenging cases***
  Different cases were presented in this popular part of the congress program and favorably managed by Dr. Nabavi.
6. ***Residents scientific competition***
  This was nicely conducted by Dr. Rahmat.

The annual neurological congress could unquestionably play an important role in promoting the status of neurology in Iran. Indeed, attendance of participants and neurologists from different parts of the world contributes to establishing a relationship between the Iranian Neurological Association and other international neurology centers across the world.

**Figure 1:**
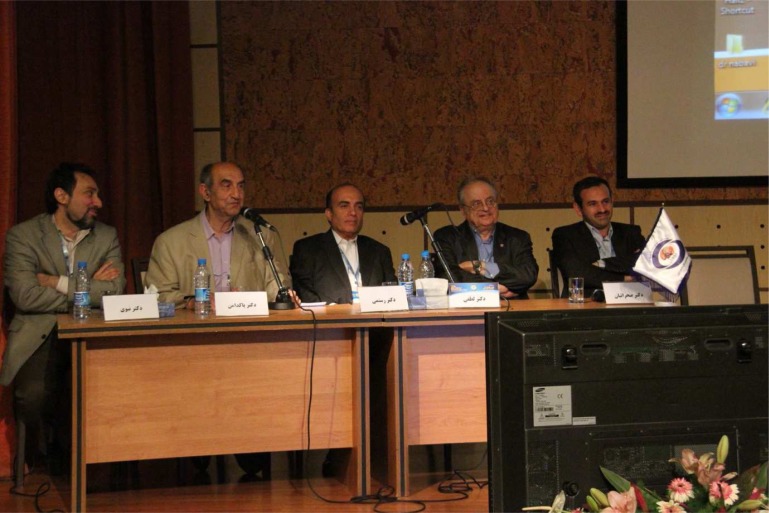
Multiple sclerosis panel: Dr. Nabavi, Prof. Pakdaman, Prof. Rostami, Prof. Lotfi, Dr. Sahraian

**Figure 2:**
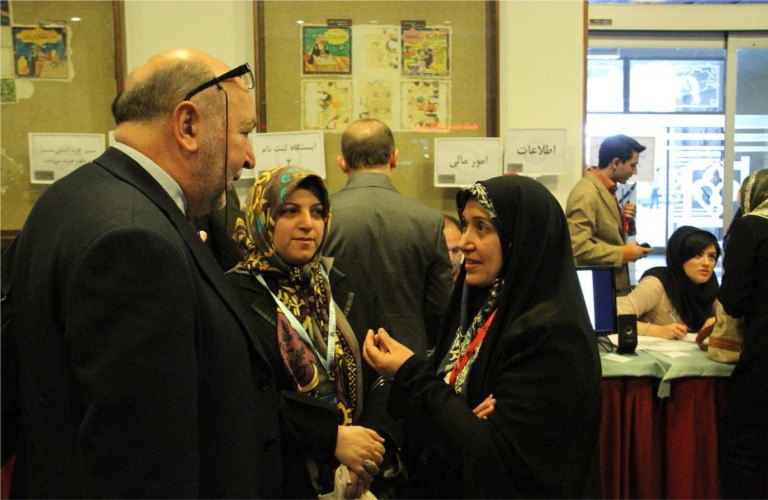
Prof. Federico, Dr. Shalbafan, Prof. Togha

